# Toward a 10 GHz High-Order Surface Acoustic Wave Resonator: A Finite-Element Study on LiNbO_3_/SiC Heterostructure Incorporating Embedded Electrodes

**DOI:** 10.3390/mi17091072

**Published:** 2026-09-09

**Authors:** Yixuan Wang, Hao Li, Qiong Wu, Tianxiang Wu, Qiaozhen Zhang

**Affiliations:** 1 College of Information, Mechanical and Electrical Engineering, Shanghai Normal University, Shanghai 200233, China1000610073@smail.shnu.edu.cn (H.L.);; 2 Key Laboratory of Modern Acoustics, MOE, Nanjing University, Nanjing 210093, China

**Keywords:** surface acoustic wave (SAW), high-order SAW resonator, embedded interdigital transducer, electromechanical coupling, finite-element analysis

## Abstract

The escalating demand for high-frequency acoustic devices in 5G/6G communications imposes stringent requirements on surface acoustic wave (SAW) resonators, including high operating frequency, large electromechanical coupling coefficient $K^{2}$, and high quality factor *Q*. However, conventional SAW devices suffer from severe trade-offs among these metrics. This work proposes and theoretically analyzes an embedded-electrode LiNbO_3_/SiC heterostructure SAW resonator tailored for high-order modes, with its frequency response evaluated via finite-element modeling. A quasi-three-dimensional periodic model consisting of LiNbO_3_/IDT/SiC structure is established, and the effects of LiNbO_3_ crystallographic orientation, normalized LiNbO_3_ thickness, and embedded-Al-electrode thickness on the resonator performance are then systematically investigated. For the selected design with Euler angle *β* = 30°, the optimal normalized LiNbO_3_ thickness is found to be $h_{\mathrm{LN}}/\lambda =0.2$. Under this crystal orientation, the optimized normalized embedded-electrode thickness is $h_{IDT}/\lambda$ = 0.06. The optimized resonator achieves a resonant frequency of *f_r_* = 12.792 GHz, a phase velocity of *V* = 12,792 m/s, a $K^{2}$ of 9.16%, and a *Q* of 1004.4. These investigation results validate the proposed LiNbO_3_/IDT/SiC heterostructure as a viable platform for pushing SAW technology into the 10 GHz regime, thereby bridging the gap between acoustic-wave devices and millimeter-wave RF systems for next-generation communications.

## 1. Introduction

The continued evolution of 5G-Advanced and prospective sixth-generation (6G) wireless systems is increasing the spectral density and frequency range that must be handled by compact radio-frequency (RF) front ends. Acoustic filters remain attractive because they combine small footprint, passive operation, high-volume manufacturability, and sharp frequency selectivity with surface acoustic wave (SAW) technology, occupying a central role in mobile communication front ends [1,2]. Extending SAW devices toward higher frequencies and wider bandwidths, however, generally requires shorter acoustic wavelengths and finer interdigital transducer (IDT) features. The resulting submicrometer-scale electrodes are increasingly difficult to fabricate reproducibly, while electrode resistance and mass loading, acoustic attenuation, parasitic modal responses, and power-induced electrode degradation become more consequential [3,4]. These limitations motivate high-frequency SAW designs that consider not only lateral scaling but also crystal orientation, layer thickness, and electrode geometry.

Several approaches have been explored to extend LiNbO_3_-based acoustic devices into the multi-gigahertz regime. Submicrometer-period LiNbO_3_ SAW transducers have demonstrated resonant frequencies of 4–12 GHz, confirming the feasibility of conventional SAW operation beyond 10 GHz while highlighting the sensitivity to IDT geometry and crystal orientation [3]. Thin-film LiNbO_3_ devices have also exploited guided and thickness-dependent acoustic modes to achieve strong coupling and wideband filtering at microwave frequencies [5,6]. Heterogeneous stacks further expand the design space by combining a piezoelectric LiNbO_3_ layer with engineered supporting layers; LiNbO_3_/SiC, LiNbO_3_/SiO_2_/Si, and LiNbO_3_/amorphous-Si/Si platforms demonstrate that substrate selection, crystal orientation, and layer thickness can strongly influence acoustic dispersion, confinement, coupling, and loss [7,8,9,10]. Recent LiNbO_3_/SiC devices have extended this approach from C-band operation toward millimeter-wave SAW resonances [10,11]. In parallel, electrode geometry and transverse modulation have been employed to suppress spurious responses [12,13]. Nevertheless, these approaches involve coupled trade-offs among frequency, electromechanical coupling, quality factor *Q*, acoustic leakage, and modal purity. Moreover, a high-frequency admittance peak alone does not establish the physical identity or confinement of the associated acoustic mode [14,15,16]. A structure-specific workflow combining broadband electrical screening, field-based mode identification, and sequential optimization is therefore needed for the embedded-electrode LiNbO_3_/SiC configuration considered in this work [17,18,19,20]. Embedded-IDT structures have been validated on quartz substrates [21], and we leverage this mature fabrication approach for the LiNbO_3_/SiC heterostructure. Building on our prior demonstration of embedded electrodes on AlN/SiO_2_/SiC [22], the present work takes a further step by integrating this electrode scheme into that same heterostructure.

In this work, an embedded-Al-electrode LiNbO_3_/SiC SAW resonator is proposed and numerically investigated using a quasi-three-dimensional periodic finite-element model in COMSOL Multiphysics 5.5. The unit cell comprises air, an embedded-Al IDT, a LiNbO_3_ piezoelectric layer, and a SiC substrate, with an acoustic wavelength of 1 µm, lateral periodic boundaries, and a perfectly matched layer beneath the substrate. Broadband frequency-domain input-admittance simulations and normalized displacement fields are first used to select an acoustically active high-frequency resonance. The Euler angle (0, *β*, 0) of LiNbO_3_ is then swept over *β*, followed by sequential parametric sweeps of the normalized LiNbO_3_ thickness and normalized Al electrode thickness. After parameter exploration, a set of optimal geometrical and angular parameters is determined for the embedded-electrode structure. Simulation yields the key performance metrics, including resonance frequency, effective electromechanical coupling coefficient *K*^2^, phase velocity *V*, *Q*, and admittance ratio AR [23,24,25]; the last quantity is derived from the periodic-cell input admittance and is not a full-filter insertion loss. These results provide a numerical design basis for high-frequency embedded-electrode LiNbO_3_/SiC SAW resonators and their subsequent experimental evaluation for future RF-front-end applications.

## 2. Modeling and Simulation

**Figure 1:**
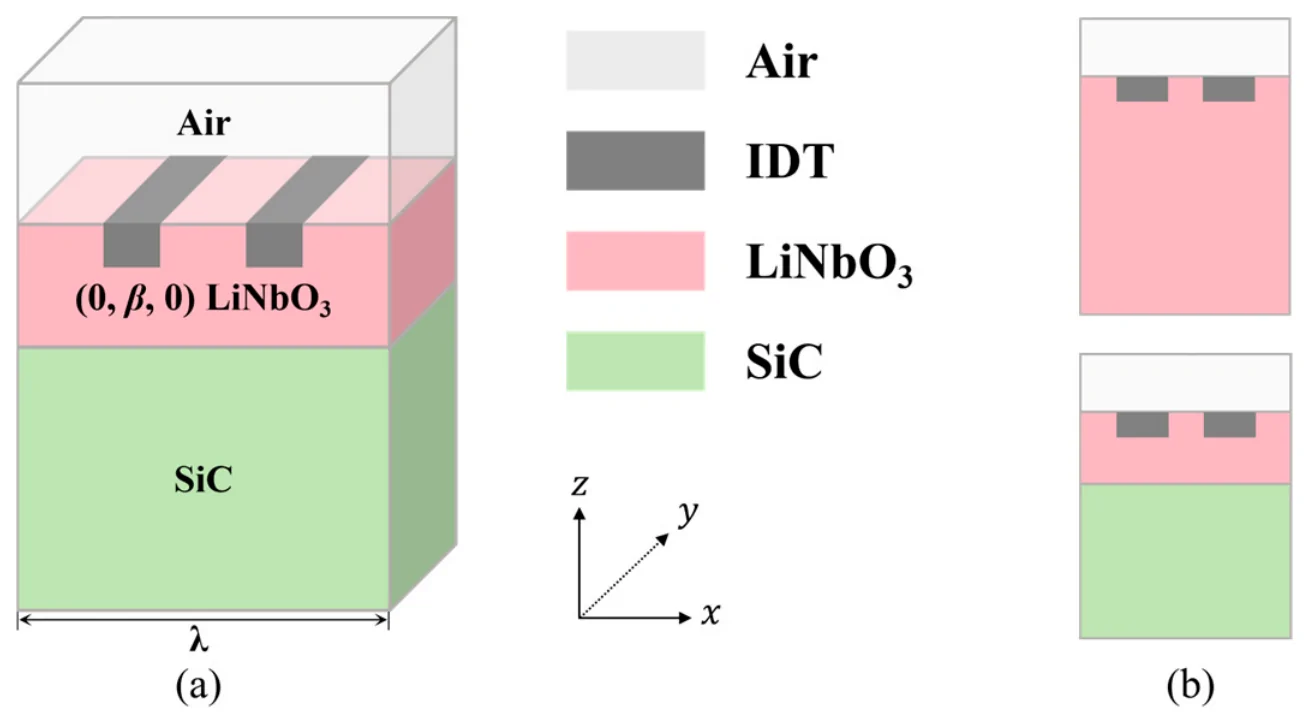
(**a**) Quasi-3D periodic finite-element model used for simulation; (**b**) 2D cross-section schematics of embedded-IDT/LiNbO_3_ structure and LiNbO_3_/IDT/SiC structure.

A quasi-three-dimensional periodic finite-element model was developed to investigate high-order surface acoustic wave (SAW) resonators based on a LiNbO_3_/SiC heterostructure with embedded-Al interdigital electrodes (IDTs). As illustrated in Figure 1a, a single periodic unit of the IDT structure was employed to simulate the periodic acoustic propagation along the device surface. Here, only plane waves propagating along the x-direction are considered, and field variation in the y-direction is neglected. Thus, periodic boundary conditions are imposed on the variables at the +y and −y surfaces [22]. This approach substantially reduces the computational cost compared with a full-device model while retaining the essential electromechanical interaction between the IDT and the acoustic field. The corresponding two-dimensional cross-sectional configurations are shown in Figure 1b, where the embedded-IDT/LiNbO_3_ structure and LiNbO_3_/IDT/SiC structure are compared. In the conventional configuration, the Al electrode is positioned directly on the upper surface of the LiNbO_3_ film, whereas in the proposed architecture, the Al electrode is embedded within the multilayer acoustic stack. The latter configuration changes the spatial relationship among the electrode, piezoelectric layer, and substrate and therefore provides an additional degree of freedom for high-order acoustic-mode engineering.

The proposed heterostructure consists of an air region, an embedded-Al IDT, a LiNbO_3_ piezoelectric layer, and a SiC substrate. The lateral periodicity of the model is determined by the acoustic wavelength λ, while the vertical dimensions of the Al electrode and LiNbO_3_ layer are normalized with respect to λ. In the initial modal screening, the normalized electrode thickness was set to *h*_IDT_/λ = 0.05, while the normalized LiNbO_3_ thickness was set to *h*_LN_/λ = 0.5. These two values were used only as the baseline parameters for identifying the target high-order mode and were subsequently varied during the structural optimization process. The acoustic wavelength used in the simulations is λ = 1 µm, corresponding to the geometric periodicity of the IDT.

The LiNbO_3_ material was modeled using its anisotropic elastic, piezoelectric, and dielectric properties, with the crystal orientation explicitly defined through the Euler-angle transformation. Because the acoustic and piezoelectric properties of LiNbO_3_ are strongly orientation dependent, maintaining a consistent crystallographic coordinate system throughout the simulations is essential for obtaining physically meaningful results. The SiC substrate was introduced as the high-velocity supporting layer, and its elastic and density parameters were included in the finite-element model. The Al electrode was modeled as an elastic conductive material to account for both its mechanical mass-loading effect and the electrical excitation of the LiNbO_3_ layer. To suppress artificial boundary reflections, a perfectly matched layer (PML) was placed at the bottom of the SiC substrate directly beneath the acoustically active region. The PML was manually constructed as a frequency-domain absorbing layer with SiC material. All structural dimensions were determined from the dominant acoustic wavelength λ at the operating frequency. The SiC substrate thickness was 4 λ (4 μm), and the PML thickness was λ (1 μm), yielding a total computational depth of 5 λ [26]. Periodic boundary conditions were applied on the lateral sides of the unit cell to mimic an infinitely repeating IDT structure. Meshing was performed according to wavelength-based criteria, with a maximum element size of λ/10.

The resonator response was calculated using a frequency-domain finite-element analysis. For each investigated structure, the input admittance was obtained from the electrical response of the IDT over the selected frequency range. The resonance frequency fr was identified from the dominant resonance feature, while the antiresonance frequency fa was determined from the corresponding antiresonance feature. The effective V and *K*^2^ were evaluated according to

(1) $$V=\frac{(f_{r}+f_{a})\lambda }{2}$$

and

(2) $$K^{2}=\frac{\pi f_{r}}{2f_{a}}\frac{1}{\tan\left(\frac{\pi f_{r}}{2f_{a}}\right)}$$

As an index to quantify the strength of the acoustic-wave response, the AR is calculated:

(3) $$AR=20\;\log_{10}\left(\frac{Y_{r}}{Y_{a}}\right)$$

where both maximum admittance $\left(Y_{r}\right)$ and minimum admittance $\left(Y_{a}\right)$ denote the admittances at the series resonant frequency $f_{r}$ and parallel resonant frequency $f_{a}$, respectively.

Here, V and $K^{2}$ were used together with the *Q* and the input-admittance response to evaluate the suitability of each investigated mode.

In the employed FEM model, the simulated *Q* is mainly governed by the prescribed mechanical and dielectric loss factors, together with other inherent electromechanical loss contributions determined by the material properties and device structure. The total loss can be expressed as

(4) $$\frac{1}{Q}=\frac{1}{Q_{\mathrm{mech}}}+\frac{1}{Q_{\mathrm{diel}}}+\frac{1}{Q_{\mathrm{other}}}$$

where *Q*_mech_, *Q*_diel_ and *Q*_other_ denote the mechanical quality factor, dielectric quality factor and other quality factor, respectively.

The exact *Q*-extraction procedure adopted in the COMSOL post-processing was implemented based on the resonant peak at the target frequency. In this work, the optimization criterion was therefore not simply the maximization of one individual parameter. Instead, the target mode was selected by considering its operating frequency, coupling strength, acoustic confinement, *Q*, and the overall cleanliness of its admittance response.

**Figure 2:**
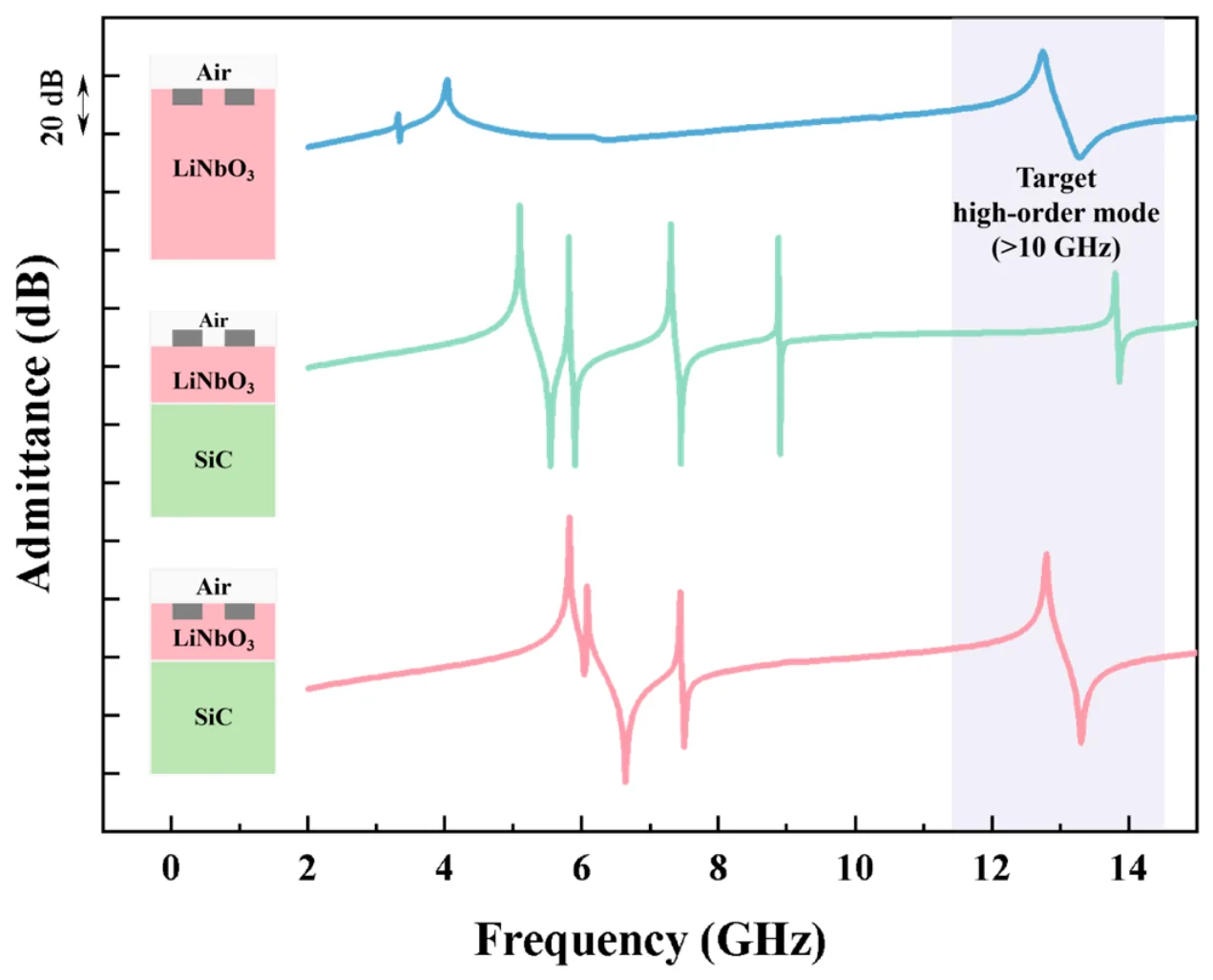
Simulated admittance responses in 2–15 GHz for three structurally different SAW resonators.

The complete optimization process was performed sequentially. First, broadband frequency-domain simulations were conducted to identify a suitable high-order mode above 10 GHz. Figure 2 shows the simulated broadband input-admittance responses for three different structures namely, the embedded-IDT/LiNbO_3_ structure, the IDT/LiNbO_3_/SiC structure, and the LiNbO_3_/IDT/SiC structure. These three configurations were evaluated under the same initial IDT geometry to investigate the effects of introducing the SiC substrate and embedded electrodes without altering the electrode layout.

As shown in Figure 2, the embedded IDT/LiNbO_3_ device exhibited significant admittance resonance in the 3–5 GHz frequency range; the IDT/LiNbO_3_/SiC structure excited multiple vibration modes in the 4–8 GHz range, while the LiNbO_3_/IDT/SiC heterostructure exhibited multiple impedance fluctuations in the 5–8 GHz range. Although the electromechanical coupling coefficients and impedance ratios of the higher-order modes in the LiNbO_3_/IDT/SiC structure are relatively low, it is noteworthy that the aforementioned embedded-electrode structures all exhibit good impedance characteristics in the 12–14 GHz frequency band, with no significant spurious modes generated. Such discrepancies prove that SiC acts beyond simple mechanical support and directly modifies the acoustic modal spectrum. Its high stiffness and acoustic velocity reshape the boundary conditions for the LiNbO_3_ thin film, tuning wave dispersion and acoustic-field depth profiles. The changed peak positions and impedance characteristics illustrate that heterostructure stacking provides a viable strategy for high-frequency mode engineering. The light-green shaded area in Figure 2 marks the frequency range chosen for the following optimization work. The resonance within this band lies above 10 GHz and is selected as our target higher-order mode; it produces a well-defined admittance signature and its corresponding acoustic displacement field can be clearly identified, as described later.

**Figure 3:**
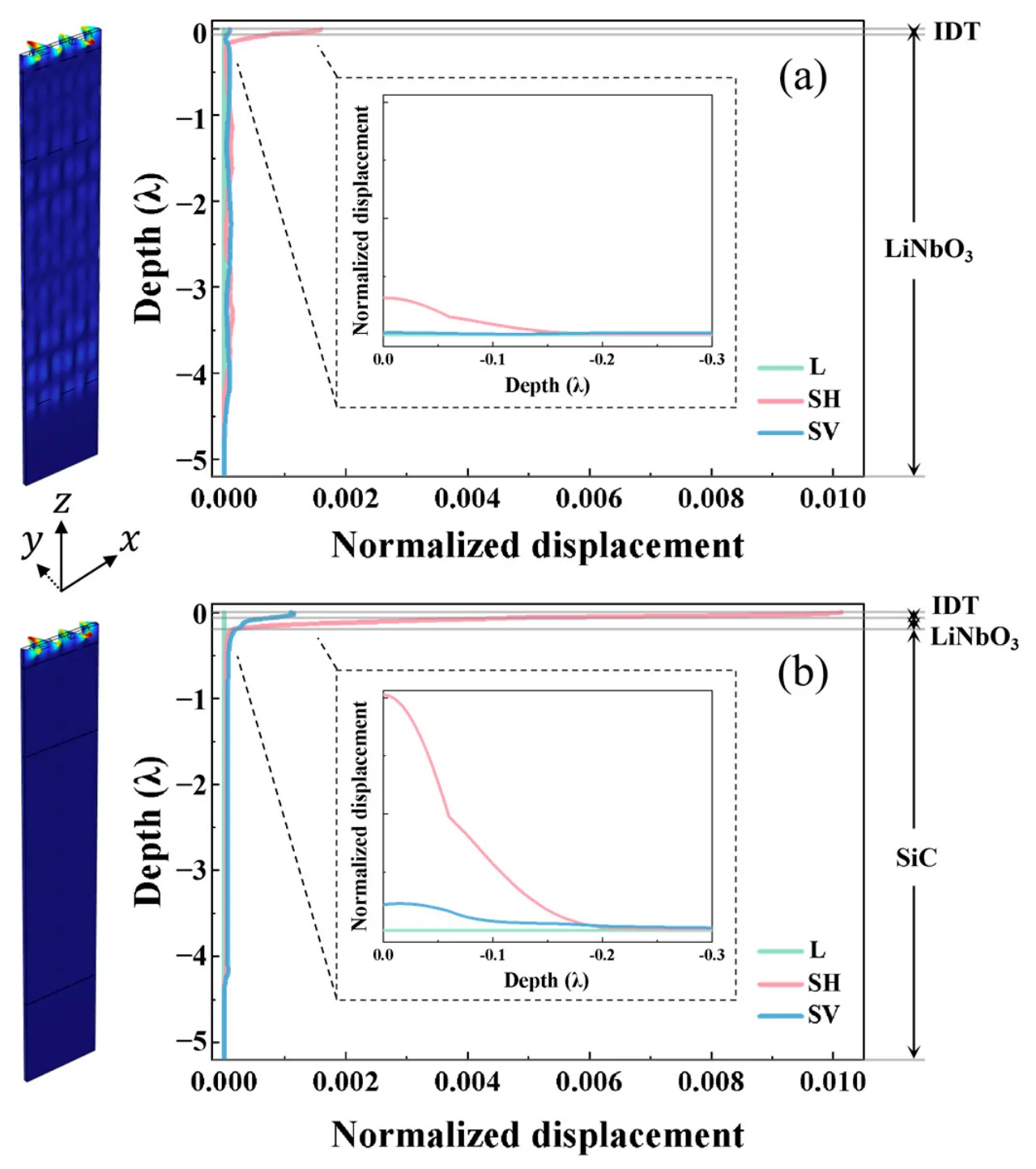
Mode shape and displacement-field distribution along the depth of the two structure configurations at their resonance frequencies, (**a**) the IDT/LiNbO_3_ structure and (**b**) the LiNbO_3_/IDT/SiC structure. The inset shows a transposed view (with the x- and y-axes interchanged) to better illustrate the exponential decay of displacement with depth.

Figure 3 presents the mode shape and depth-dependent displacement-field distributions for the two configurations at their respective resonance frequencies: (a) embedded-IDT/LiNbO_3_ and (b) LiNbO_3_/IDT/SiC. The total displacement field consists of three partial-wave contributions: longitudinal (L) wave, shear-horizontal (SH) wave, and shear-vertical (SV) wave. For the embedded-IDT/LiNbO_3_ case in Figure 3a, strong oscillatory interference between the SH and SV components is observed within the LiNbO_3_ layer, whereas the L-wave component remains nearly constant. In the SiC substrate of the LiNbO_3_/IDT/SiC heterostructure (Figure 3b), by contrast, the L, SH, and SV components maintain relatively stable amplitudes. Within the IDT region of both structures, the SH-wave normalized displacement increases; nevertheless, the amplitude enhancement of SH in panel (a) occurs over a far narrower range compared with that in panel (b).

**Figure 4:**
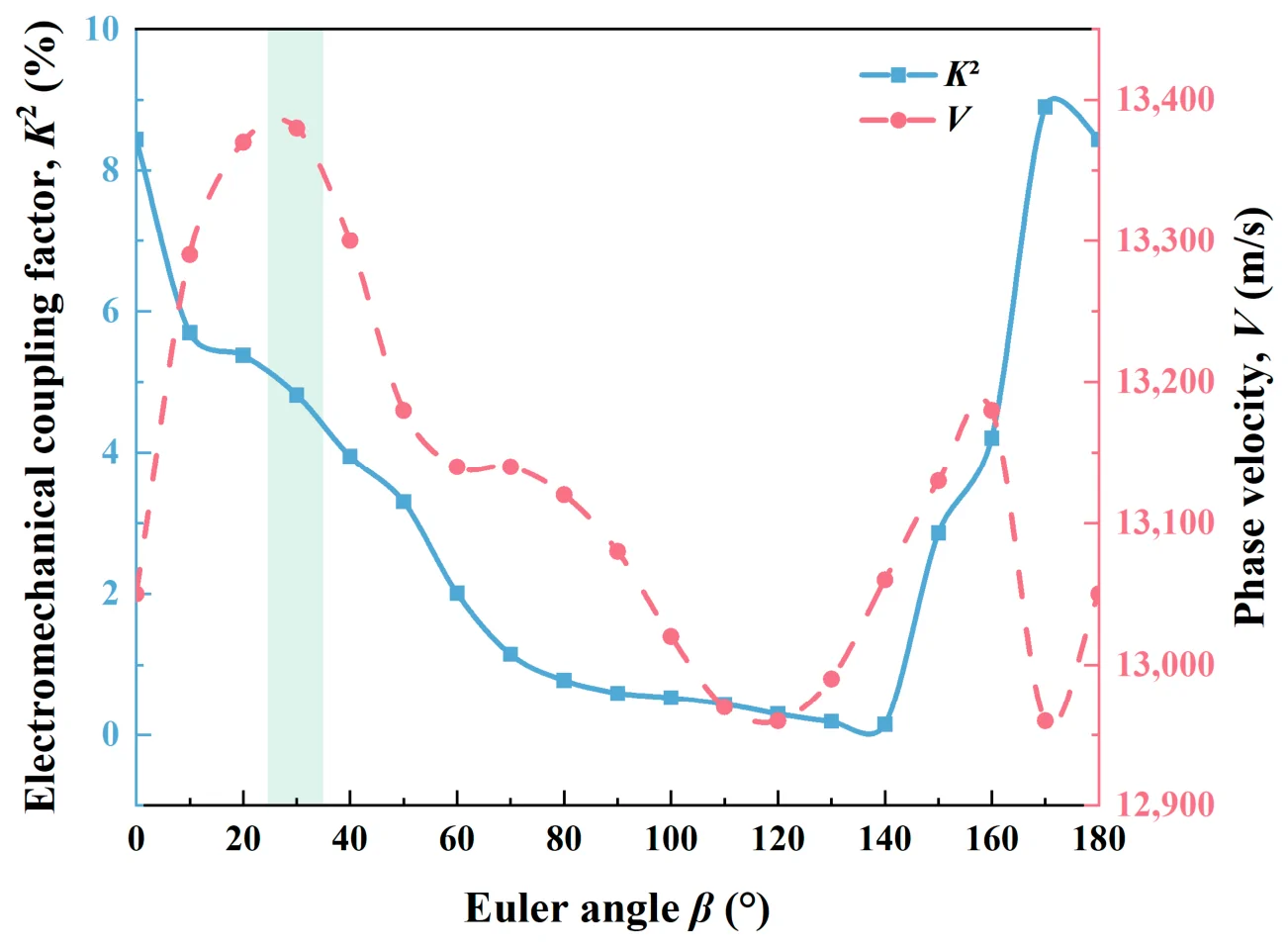
Variations in *K*^2^ and *V* as functions of *β* ranging from 0° to 180° for embedded-IDT LiNbO_3_/SiC resonator. The light-green shaded region marks the candidate orientation range for balanced performance metrics, where the final optimized Euler angle of 30° is selected.

Subsequently, the Euler angle of LiNbO_3_ was swept from 0° to 180° with a step size of 10°. Figure 4 shows the variation in *K*^2^ and *V* versus Euler angle *β* for the (0, *β*, 0) orientation configuration. During this sweep, the normalized electrode thickness and LiNbO_3_ thickness were fixed at $h_{\mathrm{IDT}}$/λ = 0.05 and $h_{\mathrm{LN}}/\lambda$ = 0.5, respectively. These values correspond to the initial structural configuration and were held constant throughout the orientation sweep to isolate the influence of crystal orientation.

Both *K*^2^ and *V* exhibit a pronounced dependence on *β*, confirming that the high-order mode is strongly affected by the crystallographic orientation of LiNbO_3_. Changing the Euler angle modifies the relationship between the acoustic propagation direction and the crystallographic axes, thereby changing both the effective elastic response experienced by the acoustic wave and the efficiency of piezoelectric excitation. Therefore, the optimum orientation is not necessarily defined solely by the absolute maximum of *K*^2^. Instead, the selected angle should provide an appropriate compromise between coupling strength, acoustic velocity, and the maintenance of the target high-order mode.

The peak simulated coupling coefficient reaches $K_{\max}^{2}\;=\;$8.90% at *β* = 170°, accompanied by *V* = 12,960 m/s. Nevertheless, the angle delivering maximum coupling fails to satisfy the multi-objective constraints for the above-10 GHz high-order mode. The light-green shaded region in Figure 4 denotes the candidate orientation range balancing all performance metrics, with the final optimized Euler angle selected as β = 30°. At this crystal cut, the device achieves $K^{2}\;=\;$4.82% and V = 13,380 m/s, establishing the fixed crystal condition for subsequent thickness optimization procedures.

## 3. Results and Discussion

**Figure 5:**
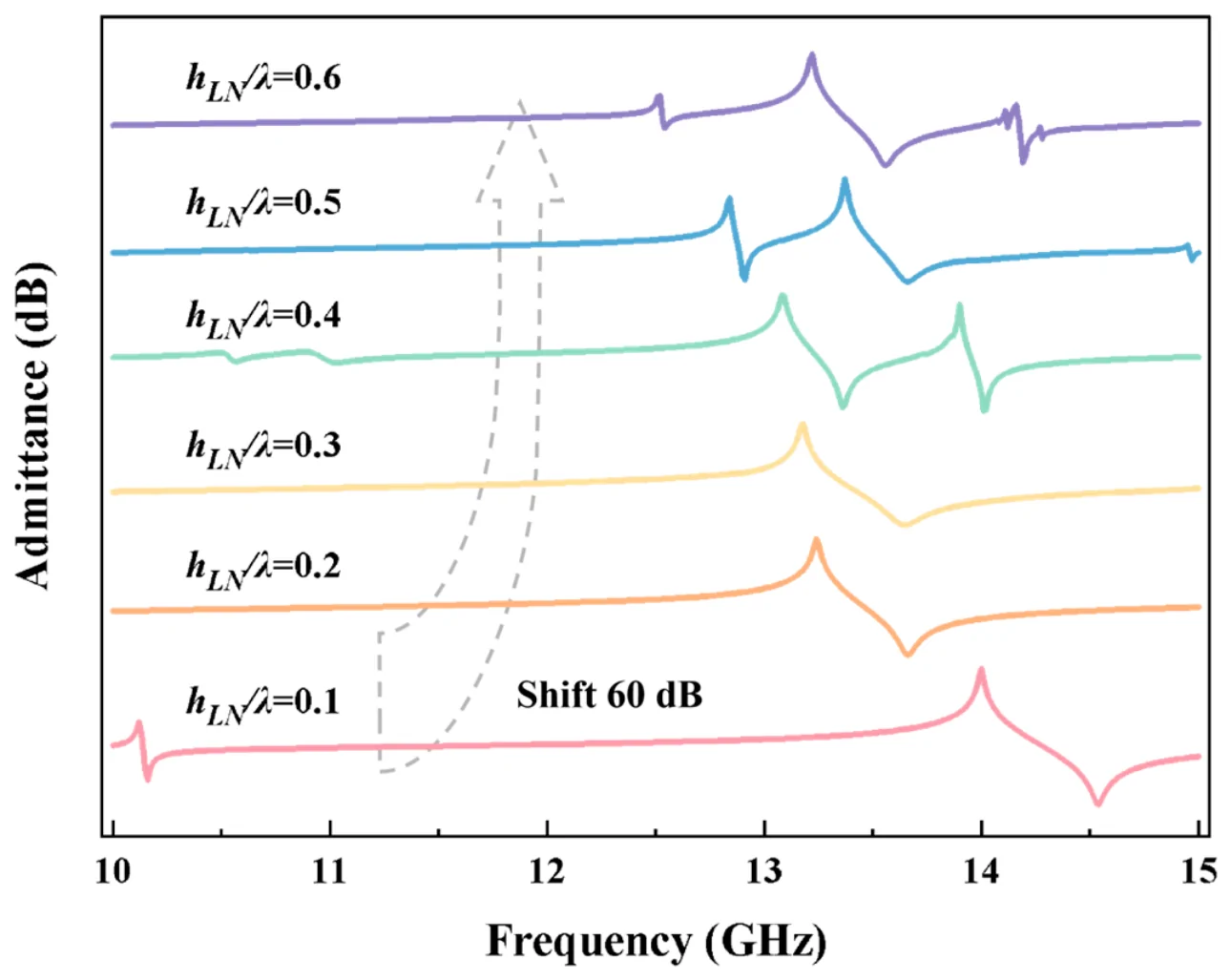
Simulated admittance curves under different normalized LN thickness $h_{\mathrm{LN}}$/λ. The Euler angle is fixed at the pre-selected value, while the electrode thickness keeps the initial baseline parameter. $h_{\mathrm{LN}}$/λ = 0.2 is determined as the optimal piezoelectric-layer thickness.

With the Euler angle fixed at the selected value, the LiNbO_3_ thickness was subsequently optimized. Figure 5 presents the simulated admittance curves obtained for different normalized LiNbO_3_ thicknesses while maintaining the initial electrode thickness at $h_{IDT}$/λ = 0.05. The normalized LiNbO_3_ thickness was varied from 0.1 to 0.6 with a step of 0.1, allowing the influence of the piezoelectric-layer thickness on the high-order resonance to be directly evaluated.

A clear dependence of the resonance response on $h_{\mathrm{LN}}$/λ can be observed in Figure 5. As the LiNbO_3_ layer’s thickness changes, the position and strength of the target resonance vary, and the corresponding admittance response changes accordingly. This behavior results from the thickness of the piezoelectric layer determining the extent to which the acoustic mode interacts with the LiNbO_3_ film and the SiC substrate. When the LiNbO_3_ layer is relatively thick, a larger portion of the acoustic field can remain within the piezoelectric material. However, excessive thickness also changes the multilayer dispersion relation and may alter the frequency separation between the target mode and neighboring modes. Conversely, when the LiNbO_3_ layer becomes too thin, the acoustic mode interacts more strongly with the underlying SiC substrate, which changes the modal distribution and may reduce the effective overlap between the acoustic field and the piezoelectric excitation region.

The simulated admittance curves demonstrate that the dependence on $h_{\mathrm{LN}}$/λ is not simply monotonic. Within the investigated range, the target mode becomes most favorable at $h_{\mathrm{LN}}$/λ = 0.2. At this optimal normalized thickness, key performance indicators are recorded as $f_{r}$ = 13.241 GHz, $f_{a}$ = 13.660 GHz, $K^{2}=\;7.34$%, V = 13,241 m/s, and Q = 688.57.

**Figure 6:**
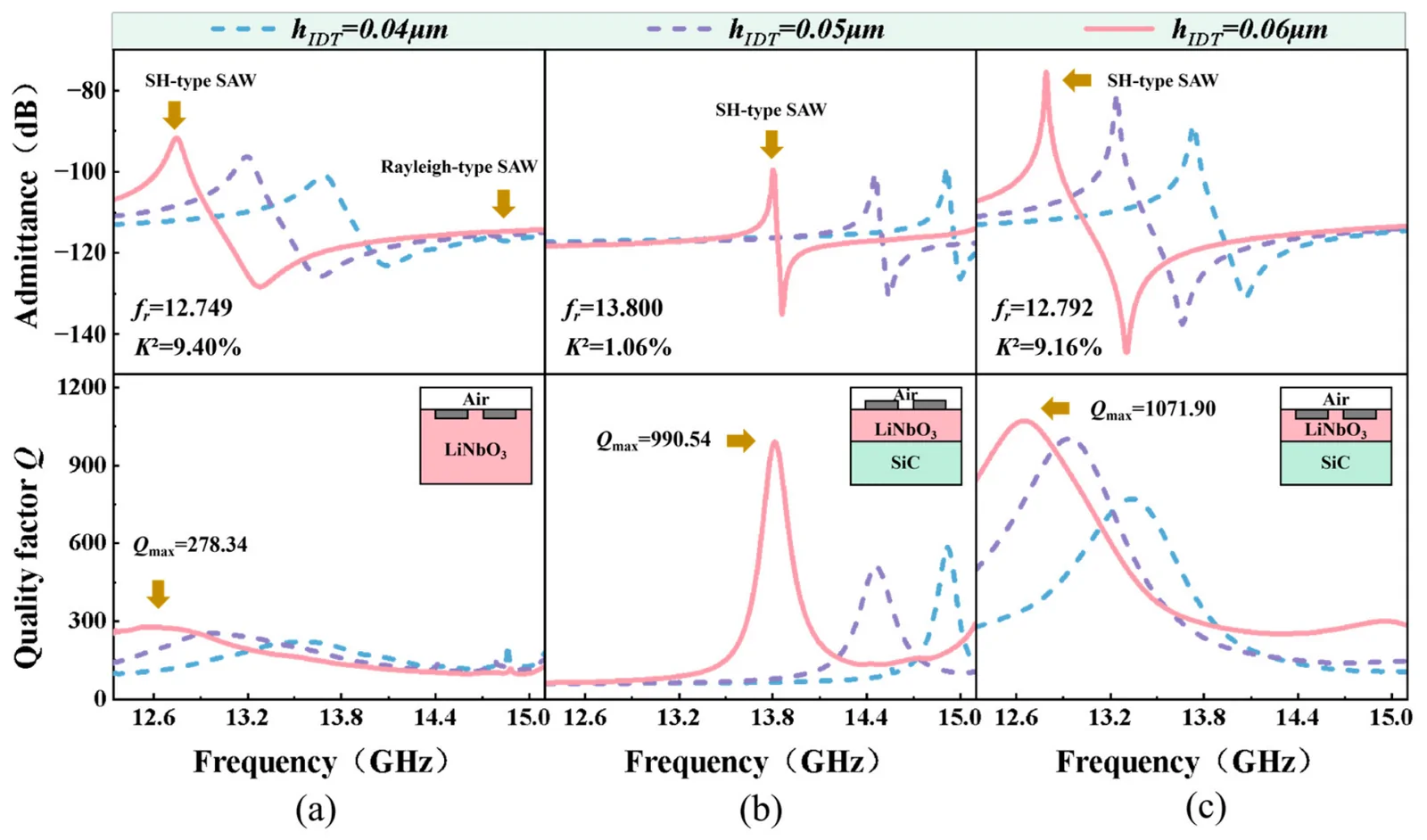
Simulated input admittance and quality-factor responses for different IDT electrode thicknesses: (**a**) embedded-IDT/LiNbO_3_ structure resonator, (**b**) IDT/LiNbO_3_/SiC structure, and (**c**) embedded-IDT LiNbO_3_/SiC heterostructure. The Euler angle and normalized LN thickness *h_LN_*/λ = 0.2 are kept constant.

The final structural parameter investigated was the Al electrode thickness. Figure 6 compares the input-admittance and quality-factor responses obtained for different electrode thicknesses in the three investigated structures: the embedded-IDT/LiNbO_3_ structure, the IDT/LiNbO_3_/SiC structure, and the proposed embedded-IDT LiNbO_3_/SiC heterostructure. In these simulations, the Euler angle was fixed at *β* = 30°, and the normalized LiNbO_3_ thickness was fixed at the optimized value $h_{\mathrm{LN}}$/λ = 0.2. Therefore, the variations observed in Figure 6 can be primarily attributed to the change in Al electrode thickness.

Furthermore, the harmonic admittances and quality-factor responses per period of infinitely periodic structures with different electrode configurations were simulated for the three structures. In this calculation, a mechanical loss (inverse of the mechanical quality factor) of 1 × 10^−4^ [27] and a dielectric loss of 0.005 [28] were adopted for the LiNbO_3_ thin film. Figure 6 shows the simulated input admittance and quality-factor responses of the acoustic wave resonators for the three structures as a function of normalized IDT thickness $h_{IDT}$/λ. In this case, a parametric sweep of $h_{IDT}$/λ was carried out from 0.02 to 0.1 with a step of 0.01. For ease of comparison, the figure also highlights the representative electrode thickness values from 0.04 to 0.06. Both the admittance magnitude and *Q* exhibit a tendency to first increase and then decrease with rising electrode thickness. As shown in Figure 6, at $h_{IDT}$/λ = 0.06, the resonator achieves the maximum admittance ratio alongside the highest *Q*. The comparison shows that the embedded-IDT/LiNbO_3_/SiC configuration provides advantages in the combined performance of *K*^2^, AR, and *Q*, indicating that the improvement is not solely attributed to the SiC substrate but also to the embedded-electrode configuration. At this optimal thickness, the admittance spectrum is free of spurious modes. The same optimization approach can be further extended to other design scenarios, enabling case-specific spurious suppression across varying frequencies and structures. Previous studies have demonstrated that nanoscale etching of LiNbO_3_ with sub-100 nm dimensions is experimentally feasible [29], supporting the fabrication feasibility of the 60 nm deep recessed grooves required for the embedded-electrode structure considered in this work. Table 1 compares the key parameters of the three optimized structures. The proposed LiNbO_3_/IDT/SiC structure exhibits superior overall performance, which can be attributed to its higher operating frequency, larger admittance ratio, and higher *Q*. It should be noted that this work mainly considers the loss from the piezoelectric layer, which is the primary loss source in the SAW resonator. Secondary contributions, including electrode resistive loss, substrate parasitic loss, and acoustic radiation loss, are omitted in the current model but will be included in future studies for a more accurate *Q* evaluation.

## 4. Conclusions

In this work, a high-order surface acoustic wave (SAW) resonator based on a LiNbO_3_/SiC heterostructure with embedded-Al electrodes is proposed and systematically investigated. A sequential optimization of the crystal orientation, LiNbO_3_ thickness, and Al electrode thickness reveals that these three parameters play distinct and non-equivalent roles: the Euler angle governs the anisotropic piezoelectric coupling, the LiNbO_3_ thickness controls acoustic energy confinement between the piezoelectric film and the SiC substrate, and the electrode thickness modulates both electrical excitation and mechanical loading. The optimized structure, with *β* = 30°, normalized LiNbO_3_ thickness $h_{\mathrm{LN}}$/λ = 0.2, and normalized Al electrode thickness $h_{IDT}$/λ = 0.06, yields a high-order SH-type SAW with resonance at 12.792 GHz, with *V* = 12,792 m/s, *K*^2^ = 9.16%, *Q* = 1004, and admittance ratio AR = 69.06 dB, along with a clean single-mode response free of spurious peaks. Collectively, this work establishes a systematic design framework that goes beyond conventional frequency scaling by synergistically integrating mode identification, crystallographic orientation engineering, piezoelectric-layer thickness optimization, and embedded-electrode design. The proposed LiNbO_3_/IDT/SiC architecture thus provides a versatile platform for balancing acoustic velocity, coupling, confinement, and *Q* at 10 GHz and beyond, offering a solid numerical foundation for future device fabrication and integration into millimeter-wave RF acoustic systems.

## Figures and Tables

**Table 1:** Comparison of the key performance parameters of the three optimized SAW resonator configurations (λ = 1 µm).

Structure	*f_r_* (GHz)	*K*^2^ (%)	Admittance Ratio (dB)	*Q*
embedded-IDT/LN	12.749	9.40	36.67	270.6
IDT/LiNbO_3_/SiC	13.800	1.06	35.45	978.1
embedded-IDT LN/SiC	12.792	9.16	69.06	1004.4

## Data Availability

The original contributions presented in this study are included in the article. Further inquiries can be directed to the corresponding author.
